# Palliative care symptoms, concerns and well-being of older people with frailty and complex care needs upon hospital discharge: a cross-sectional study

**DOI:** 10.1186/s12904-022-01065-5

**Published:** 2022-10-06

**Authors:** Kim de Nooijer, Nele Van Den Noortgate, Peter Pype, Lieve Van den Block, Lara Pivodic

**Affiliations:** 1 End-of-life Care Research Group, Vrije Universiteit Brussel (VUB) & Ghent University, Laarbeeklaan 103, 1090 Brussels, Belgium; 2 Department of Geriatric Medicine, Ghent University Hospital, Corneel Heymanslaan 10, 9000 Ghent, Belgium; 3 Department of Public Health and Primary Care, Ghent University, Corneel Heymanslaan 10, 9000 Ghent, Belgium; 4 Department of Clinical Sciences, Vrije Universiteit Brussel (VUB), Laarbeeklaan 103, 1090 Brussels, Belgium; 5 Department of Family Medicine and Chronic Care, Vrije Universiteit Brussel (VUB), Laarbeeklaan 103, Brussels, Belgium

**Keywords:** Older people, Frailty, Palliative care, Symptoms, Patient discharge, Transitions

## Abstract

**Background:**

Little is known about the nature and intensity of palliative care needs of hospitalised older people. We aimed to describe the palliative care symptoms, concerns, and well-being of older people with frailty and complex care needs upon discharge from hospital to home, and to examine the relationship between palliative care symptoms and concerns, and well-being.

**Methods:**

Cross-sectional study using baseline survey data of a pilot randomised controlled trial. Hospital staff identified patients (≥ 70 years) about to be discharged home, with a clinical frailty score of 5 to 7 and complex needs based on physician-assessment. Patients completed structured interviews, using the Integrated Palliative Care Outcome Scale (IPOS), ICEpop CAPability measure for supportive care (ICECAP-SCM) and IPOS Views on Care quality of life item. We calculated descriptive statistics.

**Results:**

We assessed 37 older people with complex needs (49% women, mean age 84, standard deviation 6.1). Symptoms rated as causing severe problems were weakness (46%) and poor mobility (40%); 75% reported that their family felt anxious at least occasionally. Of the 17 IPOS items, 41% of patients rated five or more symptoms as causing severe problems, while 14% reported that they were not severely affected by any symptom. 87% expressed feeling supported. There was a negative correlation between symptoms (IPOS) and well-being (ICECAP); r = -0.41.

**Conclusion:**

We identified a large variety of symptoms experienced by older people identified as having frailty and complex needs upon hospital discharge. Many were severely affected by multiple needs. This population should be considered for palliative care follow-up at home.

## Background

People are living longer and many are confronted with multimorbidity and frailty [1]. It has been reported that community-dwelling older people often experience complex care needs in multiple domains in the last years of life [2, 3]. Often they are hospitalised for such needs [4, 5].

Palliative care is indicated to manage the symptoms and problems experienced by older people as they near the end of life [6]. Such needs need to be addressed by health and social care providers, in the hospital but also when these patients are discharged home. Suboptimal management of their complex symptoms and concerns may lead to negative outcomes such as readmissions to the hospital and emergency department visits [7–10].

However, little is known about the extent of older people’s complex needs in the various domains relevant to health and care towards the end of life, that are, the physical, psychological, social, and spiritual domains. Previous research concerning older people upon hospital discharge mainly focused on their clinical characteristics and physical symptoms such as level of frailty [11], functional status [12, 13], or on specific symptoms or concerns such as pain and anxiety [14]. But these data do not comprehensively cover the multidimensional needs and concerns relevant towards the end of life and their inter-relationships. Moreover, previous studies did not identify patients judged as having complex care needs, thus failing to capture an important at-risk group concerning poor health outcomes and poor well-being. A reason for this is the difficulty of obtaining patient-reported research data among older people in very poor health, who are also a potentially vulnerable population [15].

Within a recent pilot randomised controlled trial (RCT) [16], we collected extensive data on multidimensional needs and well-being of older people upon discharge from the hospital. The aim of this analysis is to describe the palliative care symptoms, concerns, and well-being of older people who are identified by clinicians as having frailty and complex care needs upon hospital discharge to their home, and to examine the relationship between palliative care symptoms and concerns, and well-being.

## Methods

### Study design

We conducted a cross-sectional study using baseline survey data from a pilot randomised controlled trial (RCT) testing the feasibility, acceptability and preliminary effectiveness of a short-term specialised palliative care service intervention for older people with frailty and complex care needs in primary care in Flanders, Belgium [16]. Data were collected from February to December 2020. The study was approved by the ethics committee of Ghent University Hospital (B.U.N. B670201941807, January 22, 2020).

### Setting and participants

We aimed to include 50 eligible patients; details on the sample size calculation are reported in the study protocol of the pilot RCT [16]. Patients were recruited at the acute geriatric department and through the geriatric liaison teams of two hospitals in Flanders, one of which one is a university hospital. The aim of multidisciplinary geriatric liaison teams is to support other hospital care staff in providing geriatric care and to provide care for patients with a geriatric profile admitted to non-geriatric units [17]. Patients were eligible for this study if they were:

- aged 70 or over,
- had a Clinical Frailty Scale score (CSF) between 5 and 7 [18],
- had one or more unresolved or complex symptoms or problems in one of the four palliative care domains as judged by their treating physician; these can include situations such as, but not limited to, complex end-of-life issues such as being ‘tired of living’, difficulties with advance care planning, mental health problems, and difficulties in communication among patients, family and professionals [19, 20],
- were admitted to a hospital and about to be discharged home, and.
- were Dutch-speaking.

The data managers (KE, AJ) and the researcher (KdN) informed all eligible hospitalised patients about the study. Those patients who were interested in participating in the study were asked to provide written informed consent. If a person lacked capacity to consent (according to the clinical judgement of the treating physician), the appropriate representative as specified in the Belgian Law on Patient Rights was approached [21].

### Data collection and questionnaires

The study’s data managers/researcher approached all hospitalised eligible patients for inclusion in the study, obtained informed consent, and set a date and time for the baseline measurement. Hospital staff extracted the following characteristics from the medical files of patients who had consented to participate: age, gender, clinical frailty scale score, and medical diagnosis. The researcher and data managers then visited the patient a second time to administer a structured questionnaire in interview format. For patients who lacked capacity to consent, the representative who provided informed consent participated in the assessments as a proxy for the patient, using the same questionnaires but adapted for proxy administration. Research on the measures we used (see below) showed that family carers are able to report patients’ well-being, albeit with stronger concordance for pain compared to more personal or psychological aspects [22, 23]. We aimed to complete these interviews right before patients were discharged home. If they were discharged earlier than we had expected, we administered the questionnaire at the patients’ home. The questionnaires surveyed patient’s other socio-demographic characteristics such as living situation and educational attainment, as well as symptoms, concerns, and well-being.

To measure symptoms and concerns, we used:

- Integrated Palliative Care Outcome Scale (IPOS) [24]: includes free text responses and a structured 17-item measure of frequent palliative care needs among people with serious chronic conditions [24, 25]. Individual item scores range from 0 (absent) to 4 (overwhelming), while total scores range from 0 (minimum burden) to 68 (maximum burden) [26]. The higher the score, the greater the palliative care symptoms and concerns.

To measure well-being, we used:

- ICEpop CAPability measure for supportive care (ICECAP-SCM) [27]: a capability end-of-life measure. Patients were asked to rate aspects of well-being across seven domains: choice, love and affection, freedom from physical suffering, freedom from emotional suffering, dignity, support, and preparation. Individual attribute scores range from 1 (no capability) to 4 (full capability).
- One item of the IPOS Views on Care (VoC) measure [28]: patient’s rating of the overall quality of life on the same day. The item score ranges from 1 (very poor) to 7 (excellent).

### Statistical analysis

Descriptive statistics were used to describe the characteristics of the study population and their symptoms, concerns, and well-being. We calculated frequencies and percentages for the categorical variables, and means and standard deviations for the continuous data. We calculated Spearman correlations between palliative care needs (IPOS total scores) and well-being (ICECAP-SCM total score and IPOS VoC quality of life item score) and between the two well-being measures (IPOS VoC quality of life item score and ICECAP-SCM total score). We considered a Spearman’s r between 0 and 0.19 as very weak, between 0.2 and 0.39 as weak, between 0.40 and 0.59 as moderate, between 0.6 and 0.79 as strong and above 0.8 as very strong [29]. All analyses were performed with IBM SPSS statistical software version 27. We considered p-values lower than 0.05 as statistically significant.

## Results

In total, 145 eligible patients were approached to participate in the pilot RCT, of whom 47 consented and 37 were enrolled (10 were not enrolled due to the following reasons: patient admitted to nursing home (n = 1), patient died or was hospitalised before researcher’s visit (n = 2), not possible to approach before discharge (n = 1), not interested anymore (n = 3), concerns about COVID-19 (n = 3)). The patients who were not enrolled in the study (n = 108), were more likely to live alone than those enrolled (48% vs. 35%) but their mean age and gender proportions were comparable. Of the 37 enrolled patients, 8 patients lacked capacity to consent to participate. Their respective representative provided written informed consent and participated in the structured interviews. 57% of patients were recruited at the acute geriatrics department and the others through the geriatric liaison teams from other departments. Patients’ demographic characteristics are shown in Table 1. They were 51% male, with a mean age of 84 years. The majority were living at home with a partner/child/other (65%). 28% had cancer; among non-cancer conditions, nervous system diseases were the most prevalent category (19%).

**Table 1:** Demographic and care-related characteristics (N = 37)

Characteristics	Descriptive statistics
**Age (years)** Mean (SD) Range	83.8 (6.1) 74–98
**Gender** n(%) Female Male	18 (48.6) 19 (51.4)
**Living situation** n(%) Home, alone Home, with partner/children/other	13 (35.1) 24 (64.9)
**Clinical Frailty Score (CFS)** ^a,b^ Mean (SD)	5.8 (0.8)
**Medical diagnosis**^b^ n(%) Cancer Nervous system disease Cardiovascular disease Renal disease Respiratory disease Gastrointestinal disease Psychiatric disorder Recurrent falls Liver disease Bone fracture Other	11 (27.8) 7 (19.4) 6 (16.7) 6 (16.7) 5 (13.9) 4 (11.1) 3 (8.3) 3 (8.3) 2 (5.6) 2 (5.6) 6 (16.7)
**Number of medical diagnoses per patient n(%)** One Two Three	18 (50.0) 16 (44.4) 2 (5.6)
**Highest education completed** n(%) No education Primary education Lower secondary education Upper secondary education Higher college education	2 (5.4) 4 (10.8) 12 (32.4) 13 (35.1) 6 (16.2)
**Respondent** n (%) Patient her/himself Representative	29 (78.4) 8 (21.6)
**Location of interview** n (%) In hospital At patient’s home Not registered	9 (24.3) 27 (73.0) 1 (2.7)

SD: Standard deviation

Missing data: Medical diagnosis (n = 1), CSF (n = 2)

^a^ The CFS is scored from 0 to 9, with higher scores representing higher frailty. We recruited patients scoring 5 to 7, corresponding to ‘mildly to severely frail’

^b^ Reported by the treating physician in the hospital

### Palliative care symptoms and concerns

**Fig. 1:**
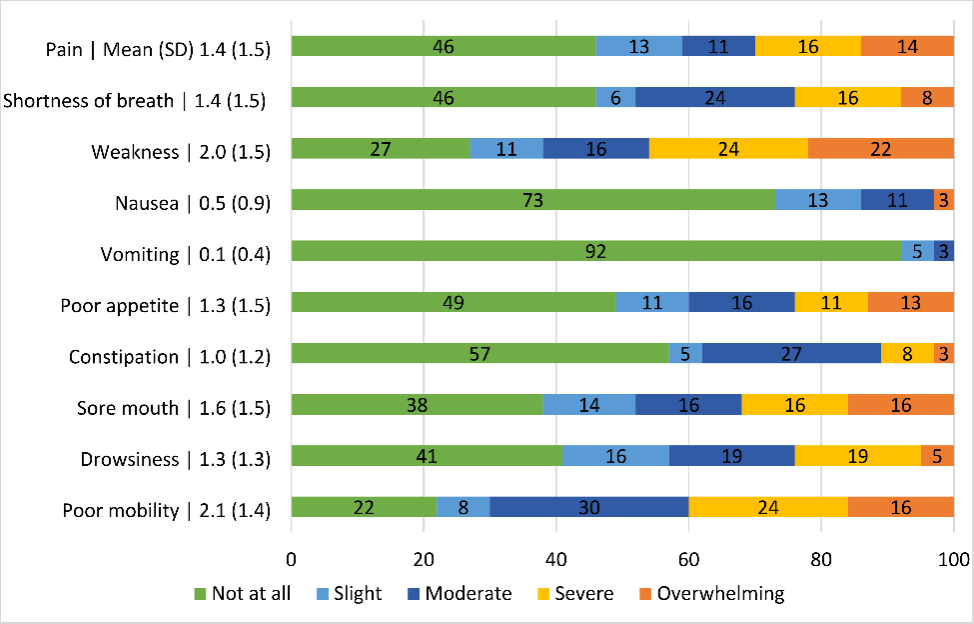
Palliative care symptoms, problems, and concerns of older people with frailty (n = 37) measured by IPOS: Mean (standard deviation) and proportion (%) Of the 17 IPOS items, 86% of the patients rated one or more symptom, problem, or concern as causing severe problems, and 41% rated five or more symptoms, problems, or concerns as causing severe problems (Table 2)

The total mean IPOS score was 21.8 (SD = 11.4) out of a maximum of 68. 73% of the patients had experienced weakness in the previous week, and 46% had experienced severe to overwhelming weakness (see Fig. 1 for details). 78% had been at least slightly/moderately affected by poor mobility, and 40% severely to overwhelmingly affected. More than half stated they had been affected by a sore mouth (62%), drowsiness (59%), pain (54%), shortness of breath (54%) and poor appetite (51%) in the previous week. Most patients were not affected by vomiting (92%) and nausea (73%). 54% had felt anxious, of whom 13% most of the time or always, and 61% had felt at least occasionally depressed in the past week. 39% of patients reported that their family had felt anxious or worried about them most or all of the time. Most patients received as much information as they wanted most or all of the time (76%). 48% said that they shared their feelings most or all of the time with their family or friends as much as they wanted and felt most or all of the time at peace (46%). 6% indicated that their problems were hardly addressed, while 64% had no problems or their problems were addressed.

**Table 2:** Number of symptoms and concerns specified as severe or overwhelming, as measured by IPOS (N = 37)

Number of symptoms/concerns by which respondents were severely or overwhelmingly affected	N (%)
0	5 (13.5)
1	5 (13.5)
2	3 (8.1)
3	5 (13.5)
4	4 (10.8)
5	5 (13.5)
6	10 (27.1)

### Well-being

**Fig. 2:**
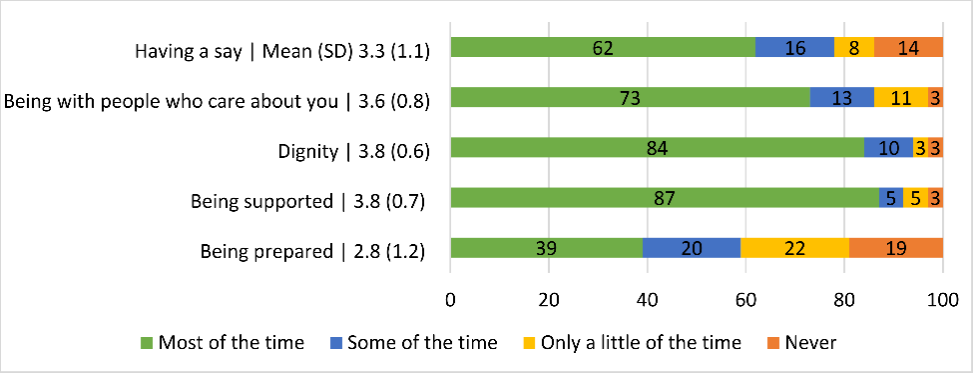
Well-being of older people with frailty (n = 37) measured by ICECAP-SCM: Mean (standard deviation) and proportion (%)

The total mean ICECAP-SCM score was 22.8 (SD = 3.9) out of a maximum of 28 (highest well-being). Between 62% and 87% of patients expressed feeling supported most of the time, able to maintain their dignity most of the time, able to be with people who care about them most of the time and being able to have a say about their life and care most of the time (see Fig. 2 for details). 35% of patients rarely experienced physical suffering and 38% indicated rarely experiencing emotional suffering. The total mean IPOS VoC quality of life item score was 4.5 (SD = 1.5) out of a maximum of 7. 32% assessed their overall quality of life in the past day with a score of 3 or lower, while 22% assessed their quality of life with a score of 6 or 7.

### Association between palliative care needs and well-being

There were moderate negative correlations between palliative care needs (IPOS total score) and well-being as measured through the ICECAP-SCM total score (Spearman’s r = -0.41; p = 0.013) and between palliative care needs (IPOS total score) and well-being as measured through the IPOS VoC quality of life item (Spearman’s r = -0.47; p = 0.003). There was a weak positive correlation between the two well-being measures (ICECAP-SCM total score and IPOS VoC quality of life item) (Spearman’s r = 0.39; p = 0.016).

## Discussion

We assessed the palliative care symptoms, concerns, and well-being of older people who were judged as having frailty and unresolved or complex needs upon hospital discharge to their homes. The data showed important heterogeneity in experienced symptom burden; some patients were severely affected by a range of symptoms and problems in multiple domains, others reported they were not severely affected by any symptom or concern. Weakness, poor mobility, sore mouth, and family anxiety were most frequently rated as causing severe problems. In terms of well-being, most patients expressed feeling supported, being able to maintain their dignity, being able to be with people who care about them and having a say about their life and care. We found that greater palliative care needs were moderately correlated with lower well-being, and we found a weak positive correlation between the two well-being measures.

Reported symptom burden upon hospital discharge varied substantially between individuals identified as having complex care needs and frailty. These findings confirm recent research that reported large heterogeneity among older people with multimorbidity and frailty in terms of their health status and symptoms and problems [30]. These findings point towards the importance of careful routine screening of each patient’s multidimensional (i.e. physical, psychological, social, and spiritual) needs as an essential part of preparing an individually tailored care response following hospital discharge to home.

Many older people who had complex care needs according to their clinician had multiple unmet palliative care symptoms and concerns upon hospital discharge. This group should be considered for palliative care follow-up at home which might include referral to specialised palliative home care services. This also has implications for the role and tasks of current specialised palliative care services. If patients are referred to these services, this is typically in the terminal phase and primarily for problems related to a cancer diagnosis [31, 32]. Adapting the work of specialised palliative care services to an older patient population with multidimensional complex needs, frailty and multimorbidity, who are not necessarily in a terminal stage of illness, may require a reorientation of their current care approach, training, and integration and collaboration with other services and models of care [33]. More research is needed to understand which palliative care structures or models are effective in addressing the complex care needs of community-dwelling older people, including identification of indicators for referral to specialised palliative home care .

Frequently reported problems and symptoms of older people in this study were pain, shortness of breath, weakness, sore mouth, drowsiness, family anxiety and depressive feelings. Comparable levels of symptom burden were reported in recent cross-sectional studies among community-dwelling older people with multimorbidity in Sweden and the UK [34, 35]. These care needs are often not well-addressed. Over the recent years increasing attention has been given to the development of evidence-based clinical practice guidelines of symptom management in older people. Some of these focus on the management of disease specific symptoms and concerns; for instance in frailty there are guidelines for sarcopenia and fatigue [36], and some on more general symptoms in older people such as pain [37]. Yet other frequent symptoms in older people towards the end of life, e.g. cachexia, still lack evidence-based best practice guidelines, especially in serious non-cancer conditions [38, 39]. There is thus an important need for development and evaluation of such evidence-based clinical practice guidelines to improve symptom control in older people with complex care needs.

We found moderate negative correlations between palliative care needs and well-being of older people with complex care needs upon hospital discharge. These results are in agreement with a previous study showing that, among older people with multimorbidity, higher levels of symptoms and concerns were associated with a lower quality of life [40]. However, the correlations were only modest, so this does not mean that all patients with high levels of symptoms and problems have low well-being. Previous qualitative studies also found that older people with multimorbidity and frailty had a relatively good quality of life and well-being [41, 42]. Based on these findings, and aligned with the action plan of the United Nations on Healthy Ageing [43], it may be particularly appropriate that healthcare providers caring for these patients not only focus on the identification and management of symptoms and concerns, but also on supporting existing abilities and capacities [41].

This study has limitations. As we recruited patients from two hospitals that were the sites of the pilot RCT from which this data were drawn, generalisability of these findings may be limited to older patients in urban areas and university hospitals. The small sample size may also compromise generalisability. Furthermore, based on our data, we cannot provide in-depth insights into inter-individual differences in symptoms and needs. While we report the number of participants interviewed in hospital versus at home (within a few days before or after discharge), we did not register the precise dates of hospital admission and discharge and hence cannot report the number of days between admission and interview. The findings of this study should be considered as a first screening of the multidimensional needs of the specific group of older people judged as having complex care needs around the time of discharge from hospital to home. As the respondents had agreed to take part in a pilot trial about a specialised palliative care intervention the sample may be have a selection bias towards those interested in palliative care (research). Also, we found that patients who declined participation were more likely to live alone. Previous research showed that people who live alone are more vulnerable to physical and psychosocial problems and symptoms [44]. Hence older people with higher symptom burden may be underrepresented in our data. This means that the already high symptom burden we found may be a lower estimate and that the actual symptom burden in this group may be even higher. Finally, the IPOS and ICECAP-SCM measures have not yet been validated specifically in the population of hospitalised older people with frailty and complex care needs. However, they have been developed and/or validated in populations with serious chronic illness, and among them older people [26, 27], and they measure multidimensional palliative care needs and well-being that are relevant for the population we studied. In the absence of other scales that measure comparable multidimensional constructs and are validated among older people with frailty, we decided to use these scales.

## Conclusion

We found large variation in the experienced symptom burden upon hospital discharge among older people judged as having frailty and unresolved or complex care needs, as well as a high and multidimensional symptom burden for many patients. This population should be considered for palliative home care follow-up which might include referral to specialised palliative care services. Greater palliative care symptoms and concerns were only moderately correlated with lower well-being, suggesting the important role of protective factors even for those affected by burdensome symptoms. Healthcare professionals should seek to identify such abilities, alongside multidimensional symptoms and concerns. This first analysis should prompt larger-scale studies to identify symptom clusters and inter-individual variation in symptoms and well-being, as well as determine the prevalence, interaction and temporal evolution of the multidimensional symptoms and concerns and well-being of older people with complex care needs, including population-based and longitudinal studies.

## Data Availability

The datasets generated and/or analyzed during the current study are not publicly available due to ethical or privacy restrictions but are available from the corresponding author on reasonable request.
