# Correction: Palliative care symptoms, concerns and well-being of older people with frailty and complex care needs upon hospital discharge: a cross-sectional study

**DOI:** 10.1186/s12904-022-01081-5

**Published:** 2022-11-26

**Authors:** Kim de Nooijer, Nele Van Den Noortgate, Peter Pype, Lieve Van den Block, Lara Pivodic

**Affiliations:** 1 End-of-life Care Research Group, Vrije Universiteit Brussel (VUB) & Ghent University, Laarbeeklaan 103, 1090 Brussels, Belgium; 2 Department of Geriatric Medicine, Ghent University Hospital, Corneel Heymanslaan 10, 9000 Ghent, Belgium; 3 Department of Public Health and Primary Care, Ghent University, Corneel Heymanslaan 10, 9000 Ghent, Belgium; 4 Department of Clinical Sciences, Vrije Universiteit Brussel (VUB), Laarbeeklaan 103, BMC Palliative Care 21, 173, 1090, 2022 Brussels, Belgium

**BMC Palliative Care 21, 173 (2022)**

10.1186/s12904-022-01065-5

Following the publication of the original article [1], the author reported that Figs. 1 and 2 were not complete as submitted.

The figures have been updated in the original article.

## References

[1] de Nooijer K, Van Den Noortgate N, Pype P (2022). Palliative care symptoms, concerns and well-being of older people with frailty and complex care needs upon hospital discharge: a cross-sectional study. BMC Palliat Care.

